# Supplementary material for: Generating 3D molecules conditional on receptor binding sites with deep generative models
Source: Chem Sci. 2022 Feb 7;13(9):2701–13. doi: 10.1039/d1sc05976a (PMC8890264; doi:10.1039/d1sc05976a)
Supplement: SC-013-D1SC05976A-s001 [file SC-013-D1SC05976A-s001.pdf]

Electronic Supplementary Material (ESI) for Chemical Science.
This journal is © The Royal Society of Chemistry 2022

Supplementary Materials: Generating 3D Molecules Conditional on Receptor Binding Sites with Deep Generative Models

Matthew Ragoza,[a] Tomohide Masuda,[b] and David Ryan Koes[c]

[a] *Intelligent Systems Program, University of Pittsburgh, Pittsburgh, PA, 15213. E-mail: mtr22@pitt.edu*

[b] *Department of Computational and Systems Biology, University of Pittsburgh, Pittsburgh, PA, 15213. E-mail: tmasuda@pitt.edu*

[c] *Department of Computational and Systems Biology, University of Pittsburgh, Pittsburgh, PA, 15213. E-mail: dkoes@pitt.edu*

Atom typing scheme. We enumerated the atoms in the CrossDocked2020 dataset and plotted their property distributions, seen in Figure S2, to create our atom typing scheme. Both for receptors and ligands, we considered element, formal charge, aromaticity, hydrogen bond acceptor/donor, and number of bonded hydrogens. We selected value ranges for the properties representative of the vast majority of the data set and used placeholders for out-of-range values. We also tried representing hydrogens explicitly. We evaluated atom typing schemes with different combinations of properties and selected the one that enabled the most accurate reconstruction of molecules. This evaluation is shown in Figure S1.

Bond inference algorithm. The goal of bond inference was to connect atoms into a single molecule with realistic bonds and hydrogens subject to atom type constraints. We first added bonds between all atoms in within a distance range. Then we set the formal charge and hydrogen count based on the atom type. Any bonds between atoms with invalid valences were removed, as well as bonds that were excessively strained in length or angle. Next the hybridization states of aromatic atoms were set to sp2, the bonds between them were set as aromatic, and the orders of all bonds were perceived using OpenBabel. Finally, empty valences were filled with either hydrogens or higher bond orders.

Atom fitting algorithm. The atom fitting algorithm jointly optimized a set of atoms and their coordinates using a reference density through an iterative approach. In each iteration, the set of grid points with the highest density were evaluated as potential new atoms to expand the current structure. The atoms were individually added to the structure and their coordinates optimized by gradient descent with respect to the reference density. If adding the new atom decreased the loss (sum of squared error), the structure was stored. At the end of each iteration, the best new structure was set as the initial structure for the next iteration, and the remaining density was set as the new reference density. This repeated until no new atoms could be found that improved the loss.

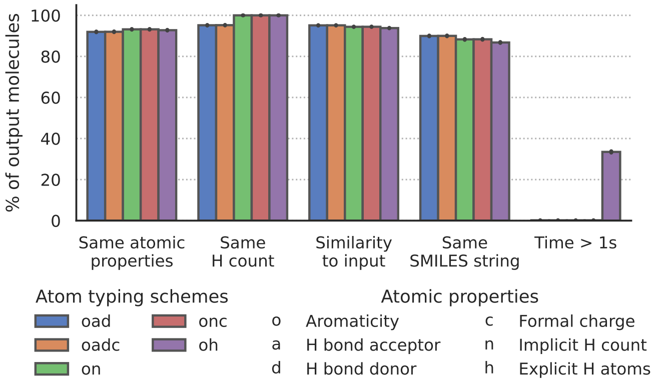

Fig. S1 Reconstructing molecules from atom types. Ability to reconstruct real molecules from their atom types and coordinates through bond inference using different atom typing schemes. We selected "oadc."

Algorithm 1: Bond inference algorithm

Data: $T \in \mathbb{R}^{N \times N_T}, C \in \mathbb{R}^{N \times 3}$
Result: $M \in$ Molecules
$M \leftarrow$ add all bonds within distance range(T, C);
$M \leftarrow$ add hydrogens and formal charges(M, T);
$M \leftarrow$ remove bad valences and geometry(M, T);
$M \leftarrow$ set hybridization and aromaticity state(M, T);
$M \leftarrow$ perceive bond orders based on geometry(M, T);
$M \leftarrow$ fill valences with hydrogen or bond orders(M, T);

Algorithm 2: Atom fitting algorithm

Data: $\mathbf{G}_{ref} \in \mathbb{R}^{N_T \times N_X \times N_Y \times N_Z}$
Result: $T \in \mathbb{R}^{N \times N_T}, C \in \mathbb{R}^{N \times 3}$
$(T, C) \leftarrow [], []$;
while found new best structs **do**
  foreach $(t_{new}, c_{new}) \leftarrow$ rank points by density$(\mathbf{G}_{ref})$ **do**
    $(T_{new}, C_{new}) \leftarrow ([T, t_{new}], [C, c_{new}])$;
    $\mathbf{G}_{diff}, C_{new}, loss \leftarrow$ gradient descent$(\mathbf{G}_{ref}, T_{new}, C_{new})$;
    if $loss$ decreased **then**
      add $(loss, \mathbf{G}_{diff}, T_{new}, C_{new})$ to new best structs;
    end
  end
  $(loss, \mathbf{G}_{ref}, T, C) \leftarrow$ new best struct;
end

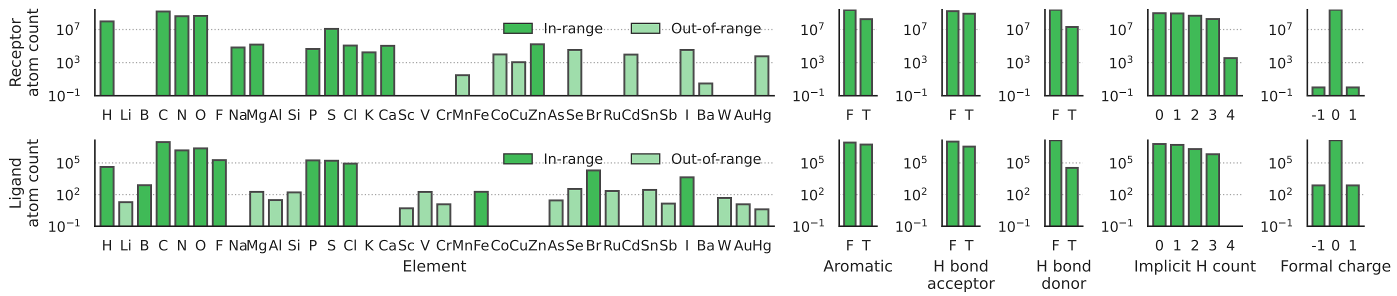

Fig. S2 Atomic properties in the CrossDocked2020 data set. Log-scale distributions of atomic properties in the CrossDocked2020 data set that were used to select value ranges represented in our atom type scheme (In-range). To limit the size of the density grids, rare elements were replaced with a placeholder element (Out-of-range). We used different elements for receptor and ligand atoms, but all other properties used the same ranges.

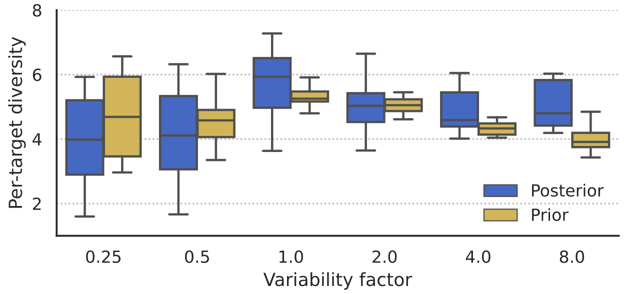

Fig. S3 Diversity of generated molecules. Comparison of two-dimensional diversity of generated molecules for a given protein binding pocket with respect to the variability factor. The diversity was computed as the inverse of the expected Tanimoto fingerprint similarity of generated molecules conditioned on each receptor.

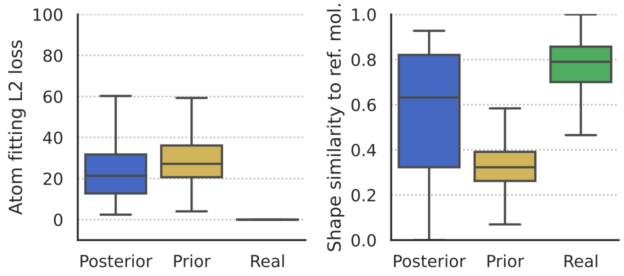

Fig. S4 Shape similarity metrics. This figure shows shape similarity metrics for generated molecules. On the left is the L2 loss of the density representation of generated molecules with the generated density to which the molecules were fit using atom fitting (i.e. the objective function value minimized by atom fitting). On the right is the Tanimoto shape similarity between generated molecules and the reference molecule.

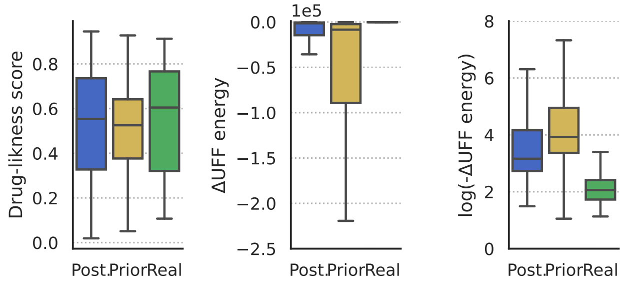

Fig. S5 Drug-likeness and change in internal energy. This figure displays quantitative estimate of drug-likeness scores for generated and real molecules.[52] In addition, the change in energy due to UFF minimization is shown for real and generated molecules in linear and logarithmic scales.

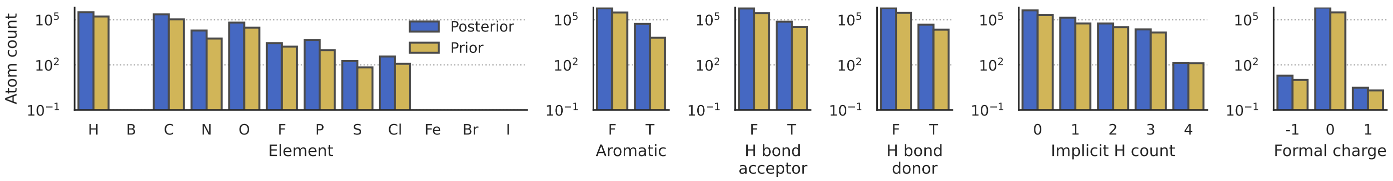

Fig. S6 Atomic properties in generated molecules. Log-scale distributions of atomic properties in molecules sampled from the generative model posterior and prior distributions.

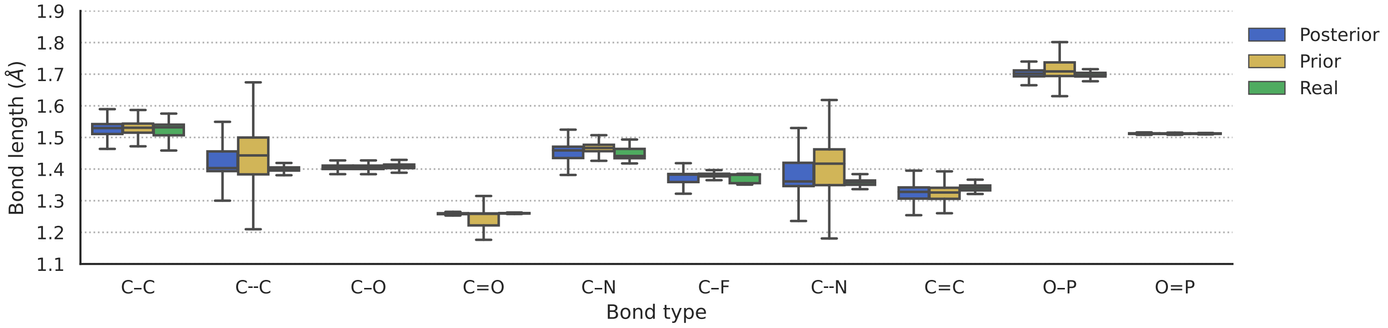

Fig. S7 Bond length distributions. Comparison of distributions of bond lengths for the ten most common bond types in the data set. For both real and generated molecules, the bond lengths are shown after UFF minimization. The bond types are indexed by the elements, bond order, and aromaticity of the bonded atoms (aromatic bonds are indicated by a dashed line).

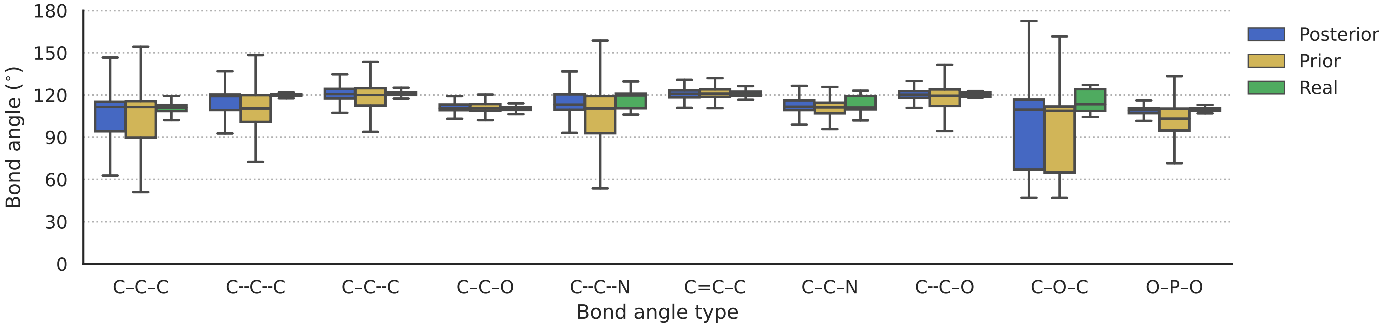

Fig. S8 Bond angle distributions. Comparison of distributions of bond angles for the ten most common bond angle types in the data set. For both real and generated molecules, the bond angles are shown after UFF minimization. The bond angle types are indexed by the elements, bond order, and aromaticity of the bonded atoms (aromatic bonds are indicated by a dashed line).

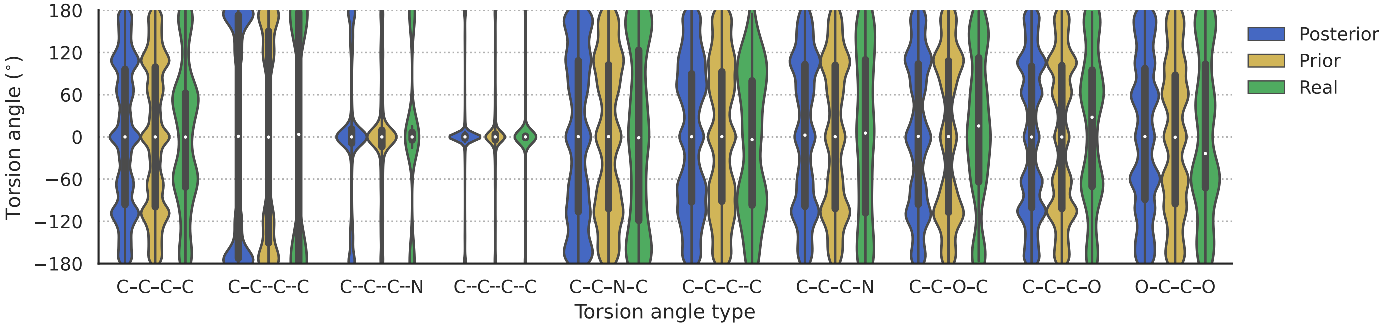

Fig. S9 Torsion angle distributions. Comparison of distributions of torsion angles for the ten most common torsion angle types in the data set. For both real and generated molecules, the torsion angles are shown after UFF minimization. The torsion angle types are indexed by the elements, bond order, and aromaticity of the bonded atoms (aromatic bonds are indicated by a dashed line).

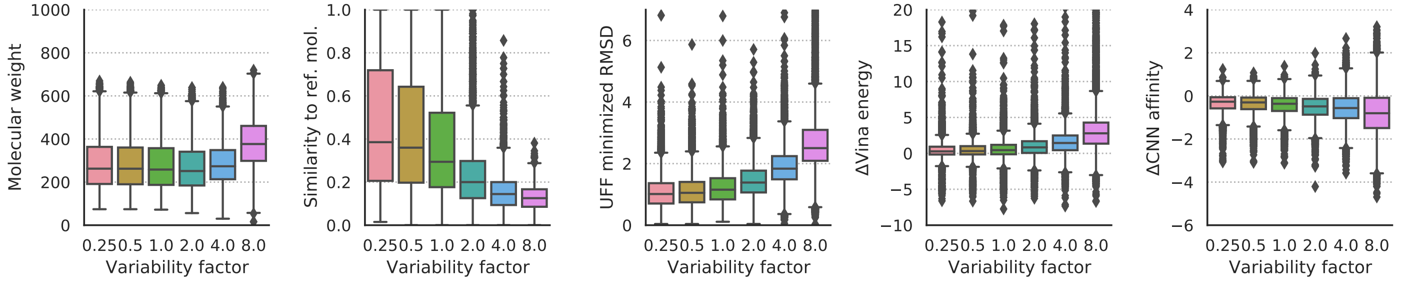

Fig. S10 Controlling variability of posterior molecules. Properties of molecules generated from the posterior with different variability factors.

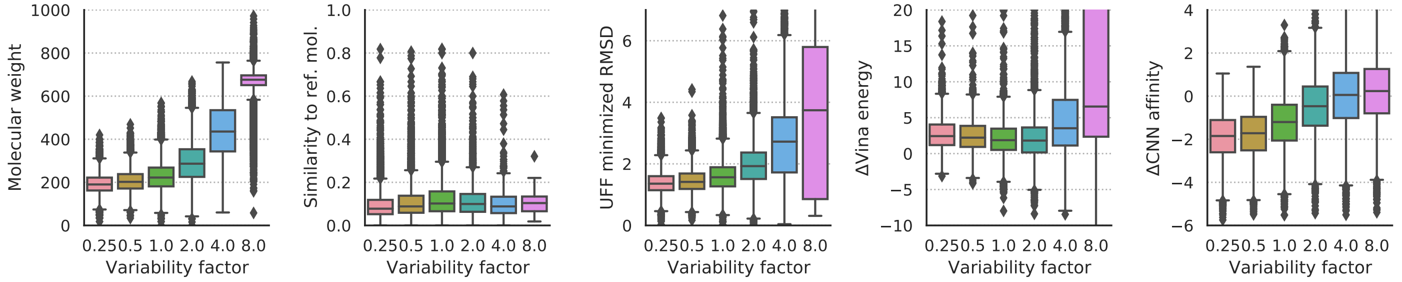

Fig. S11 Controlling variability of prior molecules. Properties of molecules generated from the prior distribution with different variability factors.

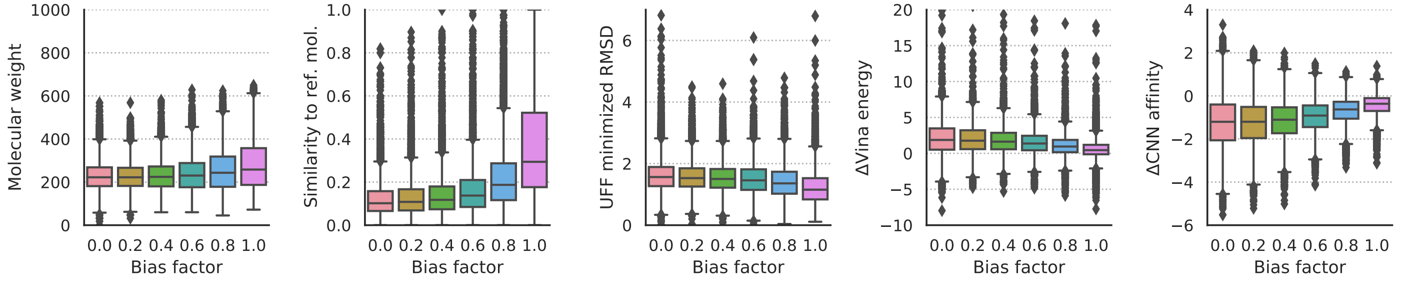

Fig. S12 Controlling bias towards reference molecule. Properties of molecules generated from distributions that were interpolated between the prior and posterior. The prior distribution corresponds to a bias factor of 0.0 and the posterior distribution corresponds to a bias factor of 1.0.

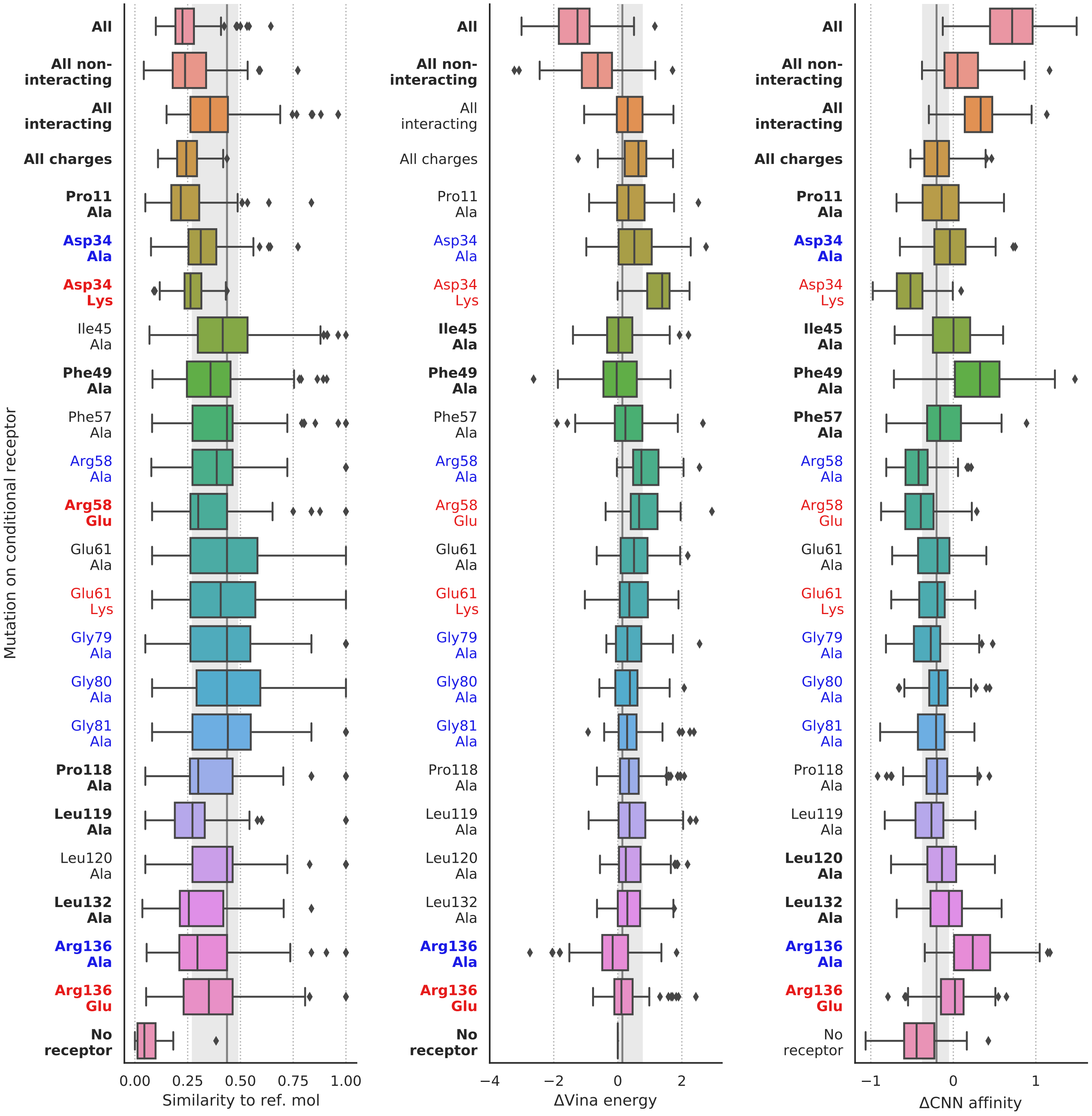

Fig. S13 Conditioning posterior molecules on mutant receptors. Properties of molecules generated from the posterior distribution when conditioned on mutant receptors. Vina energy and CNN affinity were computed with respect to the conditional (mutant) receptor. Mutated residues highlighted in blue were described in past work as interacting with the known ligand. Mutations highlighted in red inverted the charge of the residue. Gray lines in the background show the property distribution (1st, 2nd, 3rd quartile) for molecules conditioned on the wild type receptor. Mutations that caused significantly different property distributions compared to the wild type receptor are shown in bold (one-sided Kolmogorov-Smirnov test with $\alpha = 0.05$).

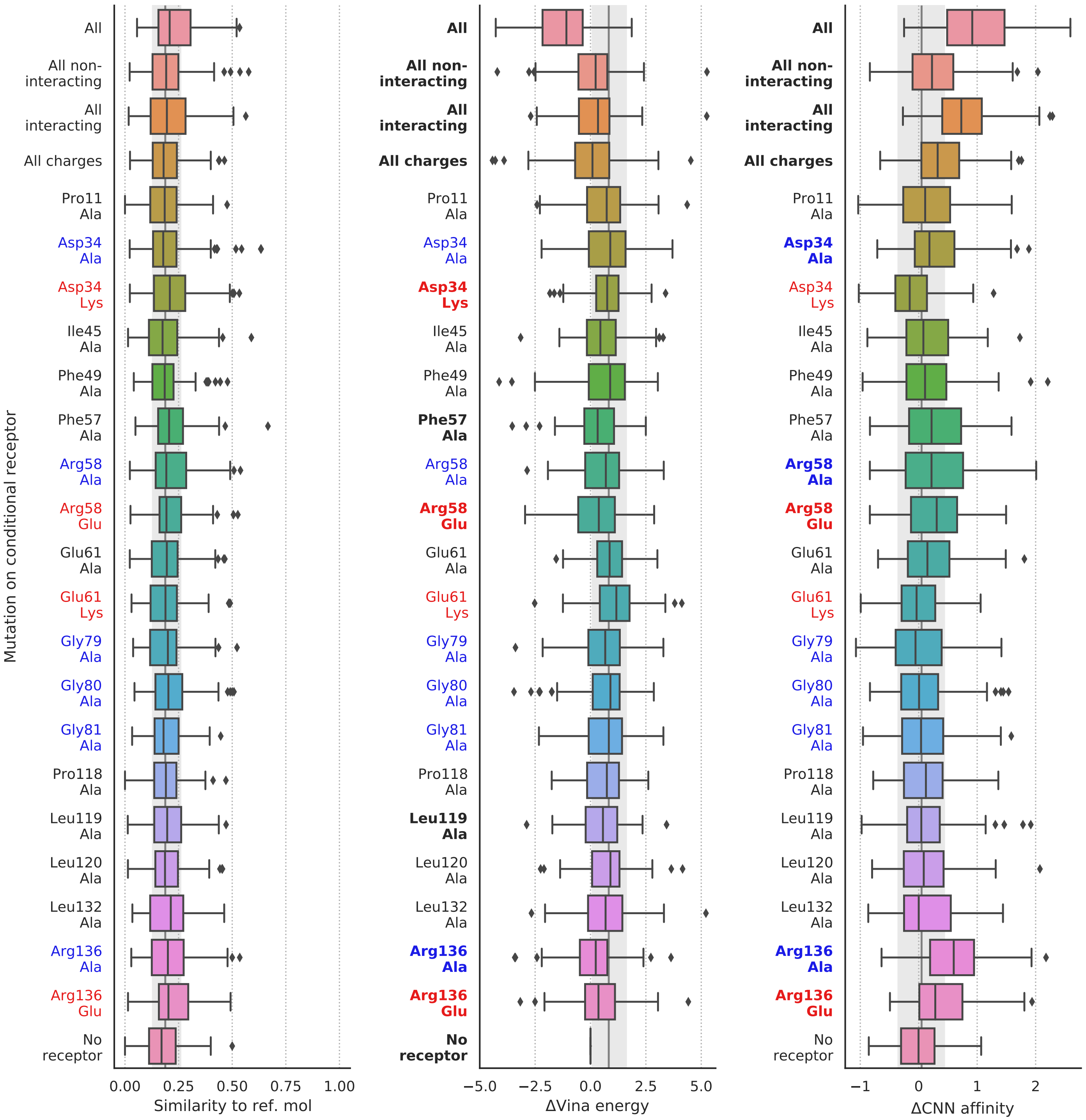

Fig. S14 Conditioning prior molecules on mutant receptors. Properties of molecules generated from the prior distribution when conditioned on mutant receptors. Vina energy and CNN affinity were computed with respect to the conditional (mutant) receptor. Mutated residues highlighted in blue were described in past work as interacting with the known ligand. Mutations highlighted in red inverted the charge of the residue. Gray lines in the background show the property distribution (1st, 2nd, 3rd quartile) for molecules conditioned on the wild type receptor. Mutations that caused significantly different property distributions compared to the wild type receptor are shown in bold (one-sided Kolmogorov-Smirnov test with $\alpha = 0.05$).
